# Exploring the Skin Benefits of Extremophilic Postbiotics from *Exiguobacterium artemiae*: A New Frontier in Thermal Protection

**DOI:** 10.3390/microorganisms13071569

**Published:** 2025-07-03

**Authors:** Haeun Lee, Dayeon Roo, Dong-Geol Lee, Seunghyun Kang, Jinwoo Min, Heecheol Kang, Young Mok Heo, Kyung Eun Lee

**Affiliations:** 1 R&I Center, COSMAX BTI, Pangyo-ro 255, Bundang-gu, Seongnam 13486, Republic of Korea; haeun.lee@cosmax.com (H.L.); dayeon.roo@cosmax.com (D.R.);; 2 R&I Center, GFC Life Science, Dongtansunhwan-daero 823, Hwaseong 18471, Republic of Korea

**Keywords:** *Exiguobacterium aurantiacum*, extremophile, heat-stress, nidogen, basement membrane, thermasome

## Abstract

Rising global temperatures increase skin exposure to heat stress, which can impair skin structure and function. While several cosmetic ingredients have been developed to mitigate heat-induced damage, most primarily aim to enhance hydration or suppress inflammation, lacking mechanistic insights into their action under heat stress. This study assessed *E. artemiae*-derived SUPER-T and its exosome form, Thermasome, in heat-stressed human skin fibroblasts. Transcriptomic profiling revealed that heat stress upregulated heat-related thermal receptors and downregulated key extracellular matrix (ECM)-related genes. Notably, treatment with SUPER-T upregulated expression of these genes, suggesting a reparative role as a barrier to alleviate heat stress at the dermal–epidermal junction. For its application in a field of cosmetics, SUPER-T encapsulated in exosomes (Thermasome) enhanced the heat resilience, suggesting its better transdermal and heat protective effects. Thermasome further improved skin heat resilience and enhanced ECM gene expression including collagen genes. Our findings provide a mechanistic basis for the development of functional cosmetical materials that target ECM remodeling under heat-stressed conditions.

## 1. Introduction

Temperature fluctuations play a crucial role as environmental factors that significantly influence the physiological functions and survival of microorganisms [1]. The survival of microorganisms depends on their ability to initiate an appropriate response to cellular stress caused by severe environmental temperature changes [2,3]. Extremophiles, microorganisms that inhabit severe environmental conditions, have developed various mechanisms to adapt to stressors such as high temperature, salinity, and acidity [4]. Among these, the *Exiguobacterium* genus is a Gram-positive bacterium with variable morphologies, from cocci to rods. The first species, *Exiguobacterium aurantiacum*, was isolated from potato effluent [5]. Strains belonging to *Exiguobacterium* have been found in various environments, such as soils; seawater; and, in particular, hydrothermal vents, which implied their heat resistance [6,7,8,9]. Such thermophilic bacteria have been observed that the stability of their proteins and the integrity of their cell membranes play crucial roles in their ability to endure thermal stress. For example, Sawle et al. examined thermal stability in thermophilic proteins using entropy and enthalpy changes at a convergence temperature, revealing that thermophiles exhibit lower enthalpic gain and higher entropic stabilization compared to mesophiles [10]. This suggests that entropic stabilization may contribute to the high melting temperatures in thermophiles, possibly due to residual structure in the denatured state [10,11]. These adaptations not only facilitate survival but also enhance the organism’s ability to function in hostile environments.

*E. artemiae* SUPER-T (KCCM13541P), isolated from hot springs, exhibits unique metabolic pathways that enable it to withstand high temperatures. Previous studies indicated that this strain showed broad temperature adaptability, allowing its survival across various thermal conditions, which can be attributed to several physiological and biochemical adaptations [12]. This unique property possibly provides benefits to skin exposed to high temperatures.

The effect of heat stress on the skin has been extensively investigated, revealing that prolonged exposure to high temperatures can lead to increased wrinkle formation, enlarged pore size, elevated sebum production, and greater skin toughness while diminishing skin brightness, elasticity, and barrier function [13]. As global temperatures continue to rise, the cutaneous response to heat stress has become increasingly important. Among different body regions, facial skin exhibits heightened thermal sensitivity, attributed to its dense distribution of thermoreceptors [14]. To mitigate the adverse effects of heat stress, various cosmetic ingredients have been explored. For instance, Aloin, a bioactive compound derived from Aloe vera, was found to have pharmacological activities against heat stress, and it protected human fibroblast cells from oxidative stress damage [15]. Similarly, a synthetic peptide, acetyl dipeptide-1 cetyl ester, alleviates the inflammatory reaction on the skin caused by the heat stress [16]. Although these ingredients are used in cosmetics, their application has largely focused on enhancing hydration and reducing inflammation, with a limited understanding of their mechanistic roles under heat-stressed conditions. Notably, heat exposure has been shown to alter the expression of basement membrane proteins, which are essential for maintaining the structural and functional integrity of the skin [17,18]. Nidogen 1 and Collagen VI alpha 1—key components of the extracellular matrix (ECM) and dermal-epidermal junction (DEJ)—are downregulated in response to heat stress. Here, we propose a novel *E. artemiae* SUPER-T-based material as a solution to protect the skin from heat-induced damage, supported by mechanistic insights into its effects on the ECM and fibroblast function.

As mentioned briefly, *E. artemiae* SUPER-T performs a unique feature in various temperatures, and based on this feature, we developed Thermal microbiome–Exosome, or “Thermasome”, a delivery system designed to encapsulate the components of this bacterium postbiotics within *Lactobacillus*-derived exosomes. Postbiotics refer to non-viable components or metabolic by-products derived from bacteria that provide health benefits to the host. These include a variety of elements such as cell-free supernatants (CFS), which are the liquid substances remaining after the removal of bacterial cells; dead bacterial cells; and metabolic by-products, such as short-chain fatty acids (SCFAs), produced during bacterial metabolism [19]. Unlike probiotics, which require live bacteria to be effective, postbiotics present notable advantages in terms of stability and safety [20,21]. Thorakkattu et al. also highlighted that postbiotics maintain substantial therapeutic potential, suggesting that they could play a significant role in promoting health and preventing disease [22]. Exosomes are nano-sized extracellular vesicles that contain mRNA, miRNA, and proteins. They engage in intracellular communication by transferring their cargo to target cells, thus promoting the proliferation of skin cells and aiding in the healing process [23]. Due to its solubility and bioavailability, several attempts have been made to encapsulate compounds within exosomes, and the encapsulation of nanoparticles has been confirmed to have higher anti-inflammatory efficacy [24].

Therefore, this research will also investigate the *E. artemiae* SUPER-T culture filtrate and Thermasome, aiming to identify optimal conditions and components that maximize its effectiveness. To comprehensively elucidate the thermal resistance mechanisms of *E. artemiae* SUPER-T, we employed RNA sequencing (RNA-seq) to analyze gene expression profiles under thermal stress conditions. In addition, in vitro assays were conducted to assess the functional capabilities of the bacterium postbiotics and Thermasome. The findings from this study will contribute to our understanding of microbial resilience in extreme environments and highlight the potential applications of thermophilic microorganisms in various biotechnological areas, including the cosmetical field.

## 2. Materials and Methods

### 2.1. Cell Culture

Human keratinocyte cell line (HaCaT) and fibroblast cell line (Hs68) purchased from American Type Culture Collection (ATCC; Manassas, VA, USA) were cultured in Dulbecco’s modified Eagle’s medium (DMEM; Hyclone Laboratories, Logan, UT, USA, #SH30243.01) supplemented with 10% fetal bovine serum (FBS; Hyclone Laboratories, Logan, UT, USA, #SH30919.03) and 1% antibiotic–antimycotic (AA; Hyclone Laboratories, #SV30079.01) in an incubator with 5% CO_2_ at 37 °C. Once cells reached their 80% confluence, cells were then trypsinized with 0.25% Trypsin-EDTA (Gibco, Billings, MT, USA, #25200-072) and used for further assays.

### 2.2. Induction of Heat Stress and Sample Treatment

We seeded 400,000 cells/well of HaCaT and 200,000 cells/well of Hs68 in a 6-well plate with 2 mL of DMEM supplemented with 10% FBS and 1% AA and incubated them for 24 h at 37 °C. After cells were adhered, the old medium was removed, and cells were treated with a blank medium (2 mL of DMEM supplemented with 1% AA) and samples. The cells with and without the samples were incubated at 41 °C for 24 h. For the negative control, cells were treated with 2 mL of DMEM supplemented with 1% AA only and incubated at 41 °C for 24 h.

### 2.3. Sample Preparation for E. artemiae SUPER-T mRNA Extraction

For the preprocessing of the RNA-sequencing, 200,000 cells/well of Hs68 were seeded in a 6-well plate with 2 mL of DMEM supplemented with 10% FBS and 1% AA and incubated for 24 h at 37 °C for cell attachment. There were three groups in this experimental design: 37 °C, 41 °C, and SUPER-T 1%. For the 37 °C group and 41 °C group, cells were treated with a blank medium only (2 mL of DMEM supplemented with 1% AA) and incubated for 24 h at 37 °C and 41 °C, respectively. For the SUPER-T 1% group, cells were treated with a blank medium with 1% of SUPER-T and incubated for 24 h at 41 °C. After incubation, cells were washed with 2 mL of DPBS once and dissolved in 1 mL of TRIzol^TM^ reagent (Invitrogen, Carlsbad, CA, USA, #15596018). Cells in TRIzol^TM^ reagent were sent to Macrogen, Inc. (Singapore) for further mRNA sequencing. The experiment was conducted using three independent samples, and all procedures were performed in triplicate to ensure the reproducibility and reliability of the results.

### 2.4. E. artemiae SUPER-T mRNA Sequencing

Total RNA concentration was calculated by Quant-IT RiboGreen (Invitrogen, #R11490). To assess the integrity of the total RNA, samples were run on the TapeStation RNA ScreenTape (Agilent, Santa Clara, CA, USA, #5067-5576). Only high-quality RNA preparations, with RIN greater than 7.0, were used for RNA library construction.

A library was independently prepared with 1 μg of total RNA for each sample by Illumina TruSeq Stranded mRNA Sample Prep Kit (Illumina, #RS-122-2101, San Diego, CA, USA). The first step in the workflow involves purifying poly-A containing mRNA molecules using poly-T-attached magnetic beads. Following purification, the mRNA was fragmented into small pieces using divalent cations under elevated temperatures. The cleaved RNA fragments were copied into first-strand cDNA using SuperScript II reverse transcriptase (Invitrogen, #18064014) and random primers. This was followed by second-strand cDNA synthesis using DNA Polymerase I, RNase H, and dUTP. These cDNA fragments then underwent an end repair process, the addition of a single ‘A’ base, and the ligation of the adapters. The products were then purified and enriched with PCR to create the final cDNA library.

The libraries were quantified using KAPA Library Quantification kits for Illumina Sequencing platforms according to the qPCR Quantification Protocol Guide (KAPA BIOSYSTEMS, Wilmington, MA, USA, #KK4854) and qualified using the TapeStation D1000 ScreenTape (Agilent Technologies, #5067-5582). Indexed libraries were then submitted to an Illumina NovaSeqX (Illumina, Inc., San Diego, CA, USA), and the paired-end (2 × 100 bp) sequencing was performed by Macrogen Incorporated.

### 2.5. Data Processing and Analysis

Paired-end sequencing reads were generated on the Illumina sequencing NovaSeqX platform. Before starting the analysis, Trimmomatic v0.38 was used to remove adapter sequences and trim bases with poor base quality. The cleaned reads were aligned to the Homo sapiens (GRCh38) using HISAT v2.1.0 [25]. The reference genome sequence and gene annotation data were downloaded from the NCBI Genome assembly and NCBI RefSeq database, respectively. Aligned data (SAM file format) were sorted and indexed using SAMtools v 1.9. After alignment, the transcripts were assembled and quantified using StringTie v2.1.3b [26,27]. Gene-level and transcript-level quantification were calculated as raw read count, FPKM (fragments per kilobase of transcript per million mapped reads), and TPM (transcripts per million).

### 2.6. Differential Gene Expression Analysis

Statistical analyses of differential gene expression were performed by DESeq2 v 1.38.3 [28] using raw counts as input. In the QC step, genes with non-zero counts in all samples were selected. PCA (principal component analysis) and MDS (multidimensional scaling) plots were generated to confirm the similarity of expression between samples. A filtered data set was applied with RLE normalization to correct the variation of library sizes among samples. The statistical significance of the differential expression gene was determined using DESeq2 nbinom WaldTest [28]. Fold change and *p*-value were extracted from the result of WaldTest. All *p*-values were adjusted by the Benjamini–Hochberg algorithm to control the false discovery rate (FDR). The significant gene list was filtered by |fold change| > 1 and raw *p*-value < 0.05. Hierarchical clustering on rlog transformed values for significant genes was performed with these parameters (distance metric = Euclidean distance, linkage method = complete). Gene-enrichment and functional annotation analysis for significant genes were carried out using gProfiler [29] against the GO (Gene Ontology) database. Adjusted *p*-values reported from the gProfiler result were derived using a one-sided hypergeometric test and corrected by the Benjamini–Hochberg method.

All data analysis and visualization of differentially expressed genes was conducted using R 4.2.2 (www.r-project.org).

### 2.7. Biocompatibility of SUPER-T and Thermasome

For the LIVE/DEAD assay, 20,000 cells of Hs68 per well of a 6-well plate were seeded and incubated for 24 h. 0.1%, and 1% of SUPER-T and Thermasome were treated with 200 μL of DMEM supplemented with 1% AA. After 24 h of incubation, the old medium was removed, and cells were washed with 200 μL of DPBS. Cells were then provided with 200 μL of LIVE/DEAD solution in DPBS. Briefly, the LIVE/DEAD solution contained 0.5 μL/mL of Calceine-AM (Thermofisher Scientific, Waltham, MA, USA, #C3099) and 2 μL/mL of Ethidium Homodimer-1 (Thermofisher Scientific, #E1169). Cells were incubated in the dark for 15 min at a 37 °C incubator. The solution was removed, and cells were washed with DPBS twice before imaging. Cells were imaged with a MICA microscope (Leica, Wetzlar, Germany, #539568). The number of live cells and dead cells was counted using ImageJ 1.54g, and the cell viability was calculated as follows:

(1) $$Cell\;Viability\left(\%\right)=\frac{Number\;of\;live\;cells}{Total\;number\;of\;cells}\times 100\%$$

For the Cell-Counting Kit-8 assay, 10,000 cells of Hs68 per well of a 96-well plate (Corning, Singapore, #3595) were seeded and incubated for 24 h. The 0.1% and 1% of SUPER-T and Thermasome were treated with 100 μL of DMEM supplemented with 1% AA. After 24 h of incubation, the old medium was removed, and cells were washed with 100 μL of DPBS. Cells were then provided with 100 μL of 10% CCK-8 solution (Dojindo, Rockville, MD, USA, #CK04-13) in DMEM supplemented with 1% AA and incubated for 2 h in the dark. The supernatant was placed into a new 96-well plate to avoid noise when measuring absorbance. The absorbance was measured at 450 nm with a multimode plate reader (PerkinElmer, Singapore, #VICTOR X3). The cell proliferation rate was calculated as follows:

(2) $$Cell\;Viability\left(\%\right)=\frac{Absorbance\;of\;sample}{Absorbance\;of\;control}\times 100\%$$

### 2.8. Real-Time Quantitative Polymerase Chain Reaction (RT-qPCR)

After induction of heat stress and treating cells with the samples (SUPER-T and Thermasome), cells were washed with DPBS once and harvested. RNA was extracted from cells using the RNeasy Mini Kit (QIAGEN, Singapore, #74116), and its concentration was measured with a spectrophotometer (Thermofisher Scientific, NanoDrop 2000/2000c) at 260 nm. Finally, 1 μL of RNA was calculated for its concentration and used for further process. RNA was denatured at 65 °C for 5 min, and cDNA was synthesized utilizing a 5X RT Master Mix (TOYOBO, Osaka, Japan, #FSQ-201). Briefly, RNA with the 5X RT Master Mix was incubated at 37 °C for 15 min, 50 °C for 5 min, and heated to 98 °C for 5 min. After the reaction, the solution was stored at 4 °C or −20 °C for further use. Quantitative real-time PCR (qPCR) was performed using a SYBR Green-based detection system. Reactions were carried out in triplicate with SYBR Green Master Mix (Thermofisher Scientific, #TFS-4368708). Amplification conditions included an initial denaturation at 95 °C, followed by 40 cycles of denaturation, annealing, and extension. The gene expression of Transient Receptor Potential Vanilloid 1 (TRPV1); Nidogen 1 (NID1); Collagen, type 6, alpha 1 (COL6A1); Collagen, type 1, alpha 1 (COL1A1); Fibrillin (FBN);Collagen, type 15, alpha 1 (COL15A1); and Collagen, type 24, alpha 1 (COL24A1) were calculated using the 2^−ΔΔCt^ method with β-ACTIN as a housekeeping gene.

## 3. Results

### 3.1. Transcriptomic Profiling and Gene Expression Analysis of E. artemiae SUPER-T

We performed mRNA sequencing to investigate the effects of thermal stress on gene expression in human skin fibroblast (Hs68) across three experimental conditions: cells cultured at physiological temperature (37 °C), cells exposed to 41 °C for 24 h to induce heat stress, and heat-stressed cells subsequently treated with the SUPER-T postbiotics. The objective of this analysis was to demonstrate the molecular consequences of thermal stress and to evaluate the transcriptional impact of the postbiotics. The sequencing output generated using NovaSeqX showed an average of 67,546,665 reads per sample, with a GC content of 49% and an AT content of 51%. Notably, approximately 96% of the reads surpassed the Q30 quality threshold, and the data integrity was considered sufficient for downstream analyses (Table 1).

**Figure 1:**
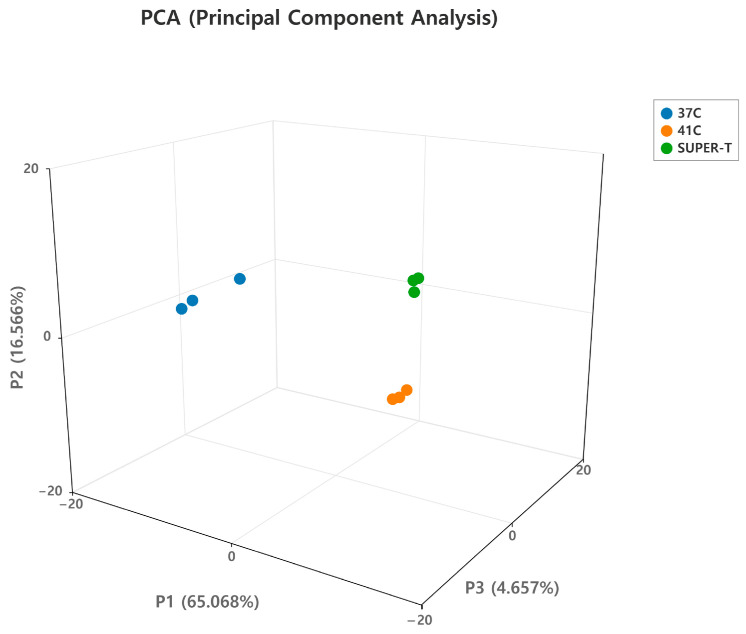
Principle component analysis (PCA) of three groups. The PCA plot illustrates the distinct tendencies among the three groups, highlighting unique changes in gene expression induced by thermal stress. Each point indicates an RNA-seq sample.

First, principal component analysis (PCA) was conducted to assess transcriptomic variation across the three conditions. The PCA plot revealed a clear separation between the 37 °C control group and the 41 °C heat-stressed group. This distinction revealed an alteration in gene expression induced by thermal stress. Notably, the SUPER-T-treated group showed distinct differences in both the heat-stressed and the control group, suggesting that postbiotics intervention regulated the heat-induced transcriptional changes in a unique way (Figure 1).

Also, pairwise comparisons between 37 °C, 41 °C, and SUPER-T groups identified genes that were differentially expressed in response to thermal stress. Using a |log_2_ fold change| threshold > 1 and an adjusted *p*-value of <0.05, a total of 3287 genes were found to have changed (Supplementary Table S1). These results reflect a strong transcriptional response to high temperatures that include both activation and inhibition of key cellular pathways.

**Figure 2:**
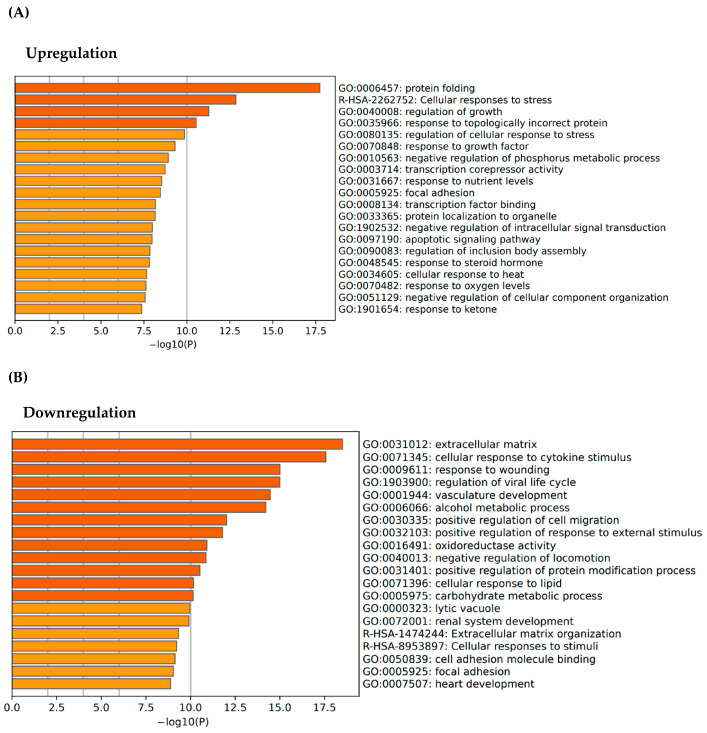
Gene ontology analysis of top 20 (**A**) upregulated and (**B**) downregulated pathways by thermal stress. Compared to the 37 °C group, the 41 °C group had increased protein folding and cellular responses to stress. In addition, the 41 °C group showed decreased gene expression related to the extracellular matrix.

To identify the biological process affected by the thermal stress, we performed gene ontology (GO) enrichment analysis for differentially expressed genes derived from the comparison between fibroblasts maintained at 37 °C, and those exposed to 41 °C for 24 h. Upregulated genes under heat stress were predominantly associated with stress response pathways, including protein folding and cellular response to heat stress (Figure 2A). These findings reflect the activation of molecular chaperone systems and proteostasis mechanisms in response to thermal stress [30,31]. On the other hand, the downregulated gene set revealed enrichment GO categories related to extracellular matrix (ECM) organization, cellular response to cytokine stimulus, and response to wounding (Figure 2B). It suggests that thermal stress not only compromises intracellular homeostasis but also significantly impairs ECM structural integrity and remodeling. The downregulation of ECM-related transcriptional mechanisms suggests that exposure to elevated temperature may impair fibroblast-mediated matrix maintenance, resulting in impaired tissue structure and function [17].

### 3.2. Regenerative Activation of ECM Pathways via SUPER-T Postbiotics

**Figure 3:**
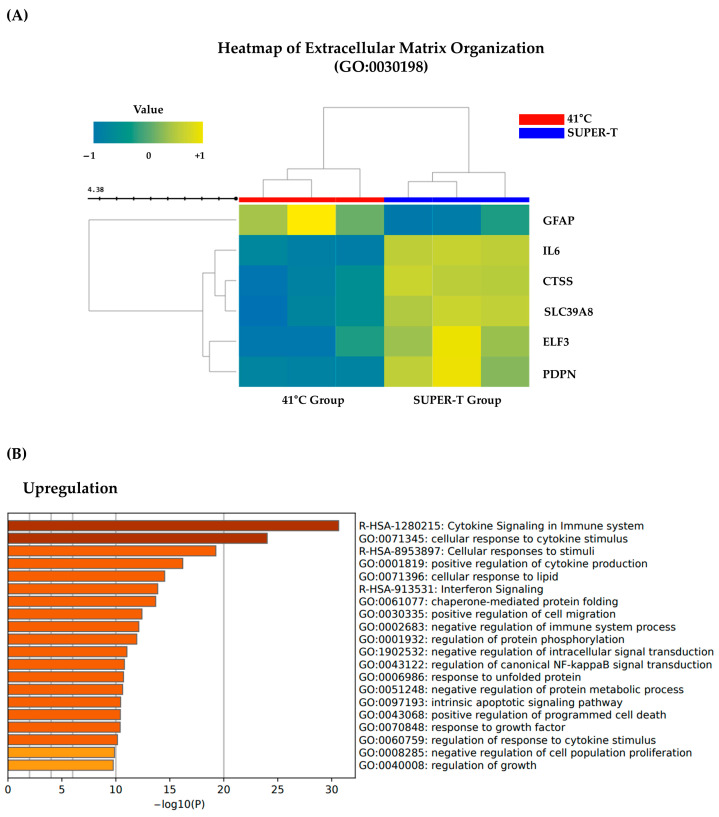
Alterations in extracellular matrix organization (ECM)-related genes by SUPER-T. (**A**) Genes involved in ECM repair and remodeling, such as IL-6, PDPN, and CTSS, were upregulated by SUPER-T treatment. (**B**) Gene ontology analysis of SUPTER-T group in comparison to 41 °C group. SUPER-T was involved in ECM remodeling by upregulating cytokine signaling and cellular response to cytokine stimulus-related genes.

Since ECM is crucial for maintaining dermal structure and mediating repair responses, we further explored whether SUPER-T postbiotics could influence ECM-related transcriptional changes. Notably, five genes involved in ECM organization and inflammatory signaling were upregulated by SUPER-T treatment, including IL-6, PDPN, CTSS, and ELF3, while GFAP was downregulated (Figure 3A).

Corresponding to these findings, a GO enrichment analysis between the 41 °C and SUPER-T groups showed a notable enrichment in cytokine-mediated signaling pathways, suggesting that SUPER-T enhances heat stress resilience in fibroblasts by activating immune-related transcriptional programs (Figure 3B).

Among the differentially expressed genes, IL-6 and PDPN were notably upregulated following SUPER-T treatment. Interleukin-6 (IL-6) is a pleiotropic cytokine involved in regulating inflammatory responses in fibroblasts and is known to affect ECM-related signaling. Notably, a recent study has demonstrated that IL-6 can induce PDPN expression in fibroblasts and basal epidermal keratinocytes, particularly under conditions of tissue damage and repair process [32,33]. Podoplanin (PDPN), a transmembrane glycoprotein involved in cell-matrix adhesion and motility, is also functionally associated with fibroblast contractility and ECM remodeling processes during wound repair [34]. The upregulation of PDPN in response to SUPER-T may thus indicate the reactivation of a matrix-interactive fibroblast phenotype suppressed under thermal-induced stress conditions.

Furthermore, the upregulation of CTSS (cathepsin S), a protease involved in the degradation of ECM components such as elastin and fibronectin, suggests that postbiotic treatment reactivates the ECM turnover. This is consistent with the state of pro-remodeling to restore matrix integrity after stress-induced damage [35].

Taken together, these transcriptional changes indicate that SUPER-T postbiotics may support the re-engagement of ECM-associated fibroblasts through IL-6-driven PDPN expression and matrix-degrading enzyme induction, contributing to the normalization of dermal environment following thermal-induced disruption.

### 3.3. Sequential Modulation of Heat Shock Protein and Collagen Genes by SUPER-T Under Thermal Stress

To further clarify the temporal regulation of stress-responsive and structural genes under thermal conditions, the expression patterns of heat shock proteins and collagen-related genes across control (37 °C), heat-stressed (41 °C), and SUPER-T-treated fibroblasts were analyzed. These gene groups were chosen to represent the key markers of cellular stress responses and ECM integrity, providing insight into how SUPER-T may influence the recovery process at the molecular level.

**Figure 4:**
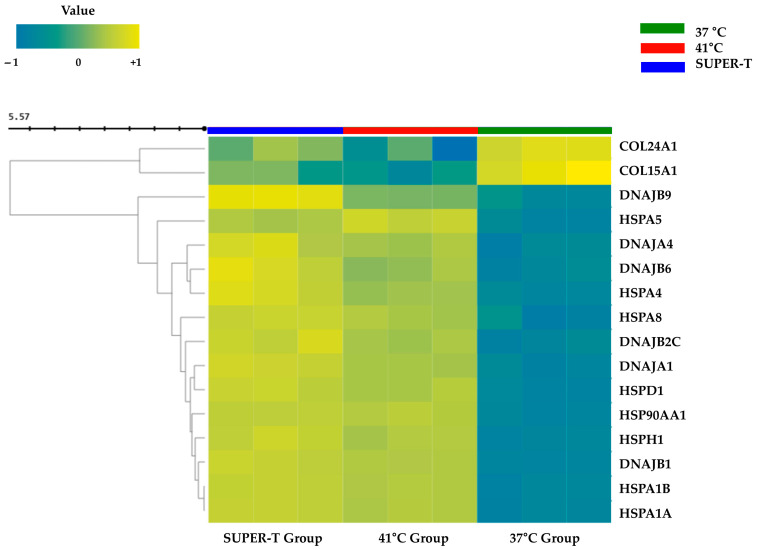
Relative heat shock protein (HSP) and collagen mRNA expression between the three groups. The effect of thermal stress and SUPER-T on the expression of heat shock proteins (HSPs) and collagen-related genes. The expression of HSPs increased at 41 °C compared to 37 °C, and SUPER-T relatively increased the expression of collagen-related genes that were significantly reduced by thermal stress.

As a result, the 41 °C group showed a marked upregulation of multiple heat shock protein (HSP) genes, including DNAJB9, HSPA5, DNAJA4, DNAJB6, HSPA4, HSPA8, DNAJB2, DNAJA1, HSPD1, HSP90AA1, HSPH1, DNAJB1, HSPA1B, and HSPA1A (Figure 4). This upregulation reflects the activation of a cytoprotective chaperone system, which plays a critical role in maintaining protein homeostasis under heat stress. Interestingly, treatment with SUPER-T further elevated the expression of these HSPs, suggesting that SUPER-T may enhance the cell’s inherent capacity to buffer heat stress. This heightened response likely represents a defense mechanism that supports sustained cellular function under prolonged or recurrent stress exposure.

In contrast, key structural genes such as COL24AL and COL15A1 were significantly downregulated by thermal stress (Figure 4), indicating a suppression of ECM biosynthesis and potential compromise of tissue structural integrity. Notably, SUPER-T treatment reversed this downregulation trend, partially restoring collagen gene expression to near-baseline levels. These findings suggest that in addition to supporting proteostasis, SUPER-T enhances the resilience of matrix-related transcriptional systems that are impaired under heat stress.

These transcriptomic patterns offer evidence to justify further validation at the functional level. Accordingly, we performed in vitro assays using primary human fibroblasts to evaluate the physiological effects of SUPER-T treatment on cell proliferation, ECM-related functions, and collagen synthesis under heat-stressed conditions.

### 3.4. Thermasome, Cosmetic Industrial Application of SUPER-T

To apply the postbiotics in a cosmetic field using industrial microbiology techniques, we used an exosome-based delivery that helps transfer active ingredients through the skin. Exosomes are vesicles that range in size from 30 to 150 nanometers and are responsible for cell-to-cell communication [36]. By making it easier for vital ingredients to reach the skin’s deeper layers, the exosome-based delivery system can boost the overall effectiveness of skincare products. Our thermophilic exosome technology was named “Thermasome.” Tangential flow filtration (TFF) was employed to isolate exosomes from the samples. The process was carried out using a membrane with an appropriate molecular weight cutoff to efficiently concentrate and purify exosome fractions. Thermasome was characterized using the ZetaView^®^ analysis system, which enables precise characterization of a wide range of bio-nanoparticles (Particle Metrix GmbH, Inning am Ammersee, Germany). The analysis revealed an average diameter of 117.4 nm and a concentration of $4.90\times 10^{5}$ particles/mL. The full width at half maximum (FWHM) was recorded at 82.2 nm, with a purity level of 89.1% (Table 2). Because of these attributes, Thermasome have established themselves as promising candidates in advanced therapeutic applications, which require extensive investigation of their functional properties.

### 3.5. Biocompatibility of SUPER-T and Thermasome

**Figure 5:**
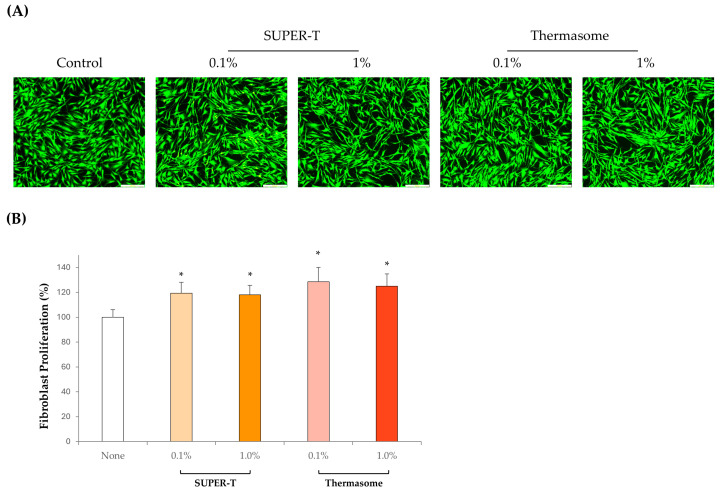
Biocompatibility of SUPER-T and Thermasome. (**A**) Cytotoxicity of 0.1% and 1% of SUPER-T and Thermasome with LIVE/DEAD assay. The scale bar was 250 μm. Live cells were stained with green color, and dead cells were stained with red colors. (**B**) The proliferation rate of human fibroblasts measured with Cell Counting Kit-8 (CCK8). * indicates * *p* < 0.05 vs. none.

Viability of fibroblasts with SUPER-T and Thermasome was measured with LIVE/DEAD assay and Cell Counting Kit-8 assay. LIVE/DEAD solution stained the live cells with calcein-AM in green and dead cells with ethidium homodimer-1 in red (Figure 5A). In all groups, cells did not show significant cell death and maintained their cell viability as there were few red-colored cells. Moreover, SUPER-T and Thermasome induced the proliferation of fibroblasts as cells cultured with the aforementioned materials performed a higher proliferation (Figure 5B). The proliferation rates of 0.1% and 1% of SUPER-T were 119.4% and 118.1%, respectively, and that of Thermasome were 128.54% and 125.0%, respectively. These results indicated that both SUPER-T and Thermasome are biocompatible at concentrations of 0.1% and 1%, with Thermasome demonstrating a slightly higher potential to promote fibroblast proliferation.

### 3.6. Effects of SUPER-T and Thermasome on the Gene Expressions of the Basement Membrane Proteins Under Thermal Stress

**Figure 6:**
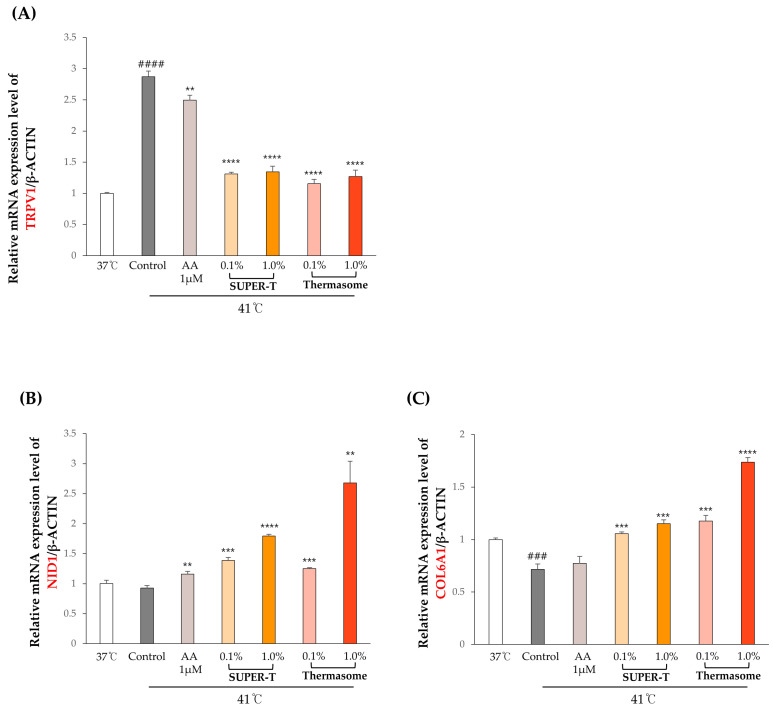

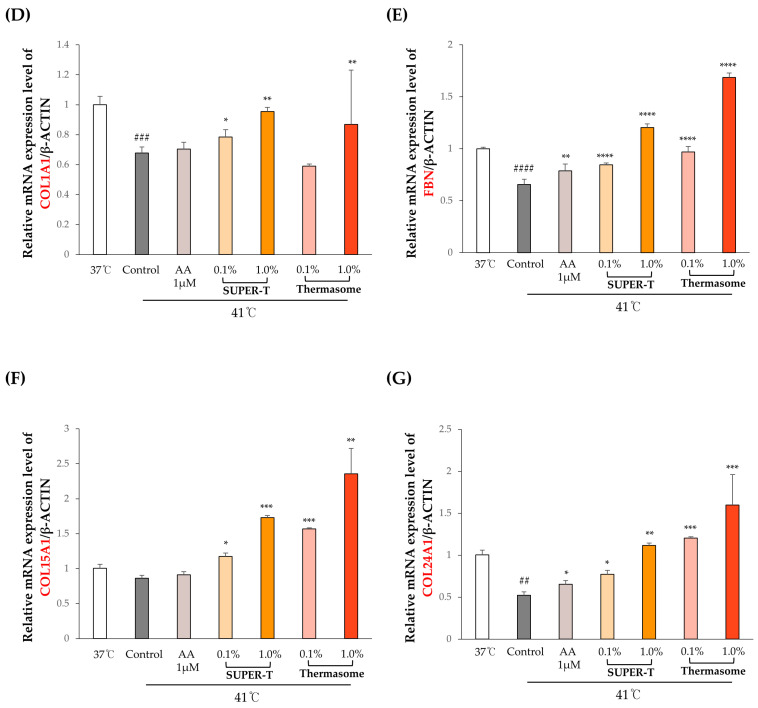
Analysis on gene expressions in HaCaT and Hs68 with 0.1% and 1% of SUPER-T and Thermasome by RT-qPCR. Relative mRNA expression levels of (**A**) TRPV1, (**B**) NID1, (**C**) COL6A1, (**D**) COL1A1, (**E**) FBN, (**F**) COL15A1, and (**G**) COL24A1 normalized to the expression level of β-actin were analyzed. We used 1 μM of ascorbic acid as a positive control. ##, ###, and #### indicate ## *p* < 0.01, ### *p* < 0.001, #### *p* < 0.0001 vs. 37 °C. *, **, ***, and **** indicate * *p* < 0.05, ** *p* < 0.01, *** *p* < 0.001, **** *p* < 0.0001 vs. control.

To evaluate the effects of SUPER-T and Thermasome on skin fibroblasts under high temperatures, we performed RT-qPCR on human dermal keratinocytes or fibroblasts treated with SUPER-T and Thermasome at concentrations of 0.1% and 1%. The expression levels of key genes involved in heat stress response and basement membrane structure were analyzed, including TRPV1, NID1, COL6A1, COL1A1, FBN1, COL15A1, and COL24A1 (Figure 6). The sequences of forward and reverse primers used in qPCR are listed in Table 3.

A transient receptor potential vanilloid 1 (TRPV1) is a heat-sensitive ion channel associated with nociceptive signaling and inflammation [37]. The result showed that the TRPV1 marker on keratinocytes was activated when cultured at a high temperature, 41 °C (Figure 6A). When cells were exposed at the same temperature but treated with SUPER-T and Thermasome, the gene expression of TRPV1 downregulated, suggesting a potential soothing effect against heat stress. Both materials at concentrations of 0.1% and 1% significantly downregulated TRPV1 expression. Moreover, their effects on fibroblasts were measured to examine the expressions of markers associated with basement membrane components, structural ECM, and specialized ECM-associated proteins.

The skin ECM is composed of a basement membrane, interstitial matrix, and elastic fiber, each of which contributes critically to the skin’s architecture and function [38]. Nidogen is a basement membrane, which is a cell-associated ECM that resides in the basement membrane of epithelial and endothelial cells [39]. Downregulation of nidogen may hinder the formation of basement membrane and hemidesmosome [18]. Upon exposure to heat, NID1 expression was downregulated, but the addition of ascorbic acid, which was a positive control, upregulated the expression (Figure 6B). The 0.1% and 1% of SUPER-T and Thermasome further enhanced the NID1 expression, and the upregulation was concentration-dependent (Figure 6B). COL6A1, which is a nonfibrillar collagen in the dermis of the skin responsible for pericellular matrix integrity, is crucial in regulating matrix assembly [40]. When fibroblasts were heat-stressed, COL6A1 expression was significantly downregulated. Treating with 0.1% and 1% of SUPER-T and Thermasome improved the upregulation of the expression; notably, Thermasome significantly upregulated the expression (Figure 6C). COL1A1 and FBN are mainly associated with elasticity, which are downregulated by heat exposure. We found that 1% of both SUPER-T and Thermasome significantly increased the gene expressions (Figure 6D,E). In accordance with RNA-seq data (Figure 4), differences in expression of COL15A1 and COL24A1 after treatment with SUPER-T and Thermasome were investigated in vitro. COL15A1 and COL24A1 are fibril-associated collagens with interrupted triple helices (FACIT) collagens, being important in ECM structure and serving as structural linkers between collagen fibrils and matrix components [41]. Our result showed that the heat exposure downregulated their expression, while SUPER-T and Thermasome alleviated and upregulated the gene expressions (Figure 6F,G). The results corresponded to the RNA-seq data.

## 4. Discussion

Heat stress is known to induce structural alterations in the skin, including degradation of collagen and damage to basement membrane proteins and the extracellular matrix. In this study, we investigated the effects of thermal stress and treatment with SUPER-T and Thermasome on the skin, focusing on molecular pathways related to skin repair. The result demonstrated that SUPER-T treatment led to a significant upregulation of the ECM-related genes, notably IL-6 and PDPN, which play pivotal roles in ECM remodeling processes. In addition, SUPER-T not only upregulated heat shock proteins but also increased the expression of extracellular matrix proteins, including NID1 and COL6A1, as confirmed by in vitro analyses. Furthermore, in vitro assays demonstrated that Thermasome further enhanced the efficacy of SUPER-T, indicating a synergistic effect on these molecular pathways.

More specifically, GO enrichment analysis revealed the upregulated genes were significantly enriched in the “cellular response to stress” and “protein folding” categories in the heat stress group, consistent with previous findings that thermal stress activates protective gene networks to maintain proteostasis [42]. In line with this, heat-stress-induced the activation of a subset of genes related to protein and heat stress responses, particularly the members of the heat shock protein family. These findings suggest that the enhanced expression of heat shock proteins and collagen-related genes could be attributed to intrinsic resistance mechanisms and cellular adaptation to thermal stress, as supported by experimental results.

Previous studies have mainly concentrated on the reduction of TRPV1 channel expression as the principal response to thermal stress in skin [43]. For example, Lee et al. proposed that ultraviolet (UV) and heat-induced matrix metalloproteinase-1 (MMP-1) might be mediated through the activation of TRPV1 in keratinocyte [44]. Also, Radtke et al. elucidated the interactions between the thermal stress and TRPV1, TRPV2, TRPV3, and TRPV4 channels [45]. In addition to the observed reduction in TRPV1 expression, our data revealed a marked upregulation of IL-6 and PDPN. Although inflammation is often perceived as a detrimental process, it is essential for the initiation of tissue repair as it promotes the removal of damaged cells and supports regeneration [46]. IL-6 is a major cytokine in early phase of inflammatory response, contributing to immune cell recruitment and the initiation of healing [47]. Deficiency of IL-6 results in delayed wound closure, reduced leukocyte recruitment, impaired angiogenesis, and diminished collagen deposition, underscoring its essential contribution to effective tissue repair [48]. The increased expression of PDPN is associated with structural remodeling of basal epithelial tissues following injury. Inadequate PDPN activity impairs cellular migration and tissue remodeling, resulting in delayed wound closure. Asai et al. reported that disruption of PDPN-CLEC-2 interaction significantly reduces keratinocyte motility during cutaneous repair, supporting a critical role for PDPN re-epithelialization [49]. In addition, a recent review indicated that PDPN expression is markedly upregulated during the proliferative phase of wound healing, where it coordinates cytoskeletal rearrangements essential for keratinocyte migration and tissue regeneration [32,50]. Moreover, CTSS is often highlighted for its involvement in inflammatory or pathological conditions, yet existing evidence underscores its physiological role in ECM reorganization following injury responses [51,52]. Collectively, these overall findings imply that microbial intervention activates a broader spectrum of biological pathways encompassing repair, remodeling, and replacement, extending beyond the focus on TRPV1 alone.

Furthermore, SUPER-T was encapsulated in exosomes to create Thermasome, with the goal of improving delivery efficiency. Exosomes have been shown to protect their cargo from enzymatic degradation and assist in transport across biological barriers, including the skin [53,54]. This may have helped preserve the activity of SUPER-T under heat stress and allowed deeper tissue penetration [53,54]. Compared to SUPER-T postbiotics, improved outcomes were observed with Thermasome in vitro which may be attributed to enhanced stability and uptake.

Exposure to high temperature resulted in a marked downregulation of key basement membrane genes, particularly NID1 and COL6A1, which are associated with dermal-epidermal junction. These changes may reflect structural deterioration of the skin fibroblasts exposed to heat. Treatment with SUPER-T and Thermasome alleviated these results, preventing downregulation of the expression of both structural and regulatory ECM components. Nidogen-1 and Collagen VI are particularly associated with linking basement membrane proteins and structurally supporting extracellular matrix [55,56,57]. The upregulation of gene expressions of Nidogen-1 and Collagen VI on human skin fibroblasts upon treatments of SUPER-T and Thermasome suggests that SUPER-T and Thermasome enhance the binding ability of basement membrane components in the epidermal–dermal junctions. Furthermore, the upregulation of COL15A1 and COL24A1 corresponded to RNA-seq results, suggesting a potential role in reestablishing dermal integrity and resilience under thermal stress. Further experiments in more physiologically relevant models would validate those materials’ properties in suppressing heat-induced skin damage.

Several considerations should be taken into account when interpreting these findings. While in vitro (cell culture) models provide relative quantitative gene expression insights, they may not fully capture the complexity of skin tissue or in vivo responses. The statistical data could be further strengthened with increased sample size or replicates. The focus on selected genes and protein expression, rather than comprehensive physiological outcomes, leaves some aspects of broader and long-term effects to be clarified. Furthermore, the validation across diverse skin conditions, age groups, and ethnicities would support the broader applicability of SUPER-T and Thermasome.

## 5. Conclusions

In this research, we introduced SUPER-T and Thermasome as efficient ingredients for restraining heat-stressed skin fibroblasts and figured out how those materials could prevent downregulation of ECM-related genes. In particular, observed upregulation of NID1 and COL6A1 suggests that SUPER-T and Thermasome may enhance the bonding strength between the epidermal–dermal sites with reduced integrity due to thermal stress by promoting their linkage. Although the complexities of thermal and thermal defense mechanisms remain largely enigmatic, the utilization of ingredients derived from extremophiles that thrive in high-temperature environments has provided a more profound insight into these intricate processes. Therefore, the current study, which examined mechanisms related to heat through the application of postbiotics derived from extreme environments, holds promise for future applications across various industries, including the cosmetic field.

## Figures and Tables

**Table 1:** RNA sequencing raw data statistics.

Sample ID	Total Read Bases (Bp)	Total Reads	GC%	AT%	Q20%	Q30%
37 °C_1	6,301,270,820	62,388,820	49.1	50.9	99	96.3
37 °C_2	7,270,146,246	71,981,646	49.4	50.6	99	96.1
37 °C_3	7,509,311,822	74,349,622	49.2	50.8	99	96.1
41 °C_1	6,868,869,408	68,008,608	49.7	50.3	99	96
41 °C_2	6,113,338,908	60,528,108	49.8	50.2	99	96.1
41 °C_3	6,094,026,294	60,336,894	49.8	50.2	98.8	95.5
SUPER-T_1	6,236,462,958	61,747,158	49.1	50.9	99	96.3
SUPER-T_2	7,324,428,696	72,519,096	49.3	50.7	98.8	95.8
SUPER-T_3	7,682,063,030	76,060,030	49.4	50.6	99	96.2

**Table 2:** Characterization of Thermasome.

Diameter/nm	Particles/mL	FWHM/nm	Purity
117.4	$4.90\times 10^{5}$	82.2	89.10%

**Table 3:** Primer sequences used in qPCR.

Gene	Forward	Reverse
*hβ-ACTIN*	CATGTACGTTGCTATCCAGGC	CTCCTTAATGTCACGCACGAT
*hTRPV1*	GTGGACAGCTACAGTGAGATGC	GGAAGCCACATACTCCTTGAGG
*hNID1*	AGGAGCTCTTTCCCTTCGGC	CGGGGGTTCACTCGTAGCAA
*hCOL6A1*	GCCTTCCTGAAGAATGTCACCG	TCCAGCAGGATGGTGATGTCAG
*hCOL1A1*	GCTTGGTCCACTTGCTTGAAGA	GAGCATTGCCTTTGATTGCTG
*hFBN*	GCGGAAATCAGTGTATTGTCCC	CAGTGTTGTATGGATCTGGAGC
*hCOL15A1*	GGACTTGGATTCGAGGATACCG	AATACTGGCTCCATCCATCCC
*hCOL24A1*	GATACCATGGAGCAGATGGC	CCCTTGTTCACCCTTTGGG

## Data Availability

RNA-seq data are available at https://www.ncbi.nlm.nih.gov/. BioProject ID PRJNA1255305 (accessed on 29 April 2025). BioSample accessions codes are as follows: SAMN48139743, SAMN48139744, SAMN48139745. Other contributions presented in this study are included in the article and Supplementary Material. Further inquiries can be directed to the corresponding authors.

## Supplementary Materials

The following supporting information can be downloaded at https://www.mdpi.com/article/10.3390/microorganisms13071569/s1. Table S1: Differentially expressed gene raw data of three groups.

## Abbreviations

The following abbreviations are used in this manuscript:

ECM	Extracellular matrix
qPCR	Quantitative polymerase chain reaction
CFS	Cell-free supernatant
SCFAs	Short-chain fatty acids
mRNA	Messenger ribonucleic acid
miRNA	Micro ribonucleic acid
RNA seq	RNA sequencing
ATCC	American type culture collection
DMEM	Dulbecco’s modified Eagle’s medium
FBS	Fetal bovine serum
AA	Antibiotic–antimycotic
NCBI	National center for biotechnology information
FPKM	Fragments per kilobase of transcript per million mapped reads
TPM	Transcripts per million
QC	Quality control
PCA	Principal component analysis
MDS	Multidimensional scaling
FDR	False discovery rate
GO	Gene ontology
DPBS	Dulbecco’s phosphate-buffered saline
CCK-8	Cell-counting kit-8
RT-qPCR	Real-time quantitative polymerase chain reaction
TRPV1	Transient receptor potential vanilloid 1
NID1	Nidogen 1
COL6A1	Collagen, type 6, alpha 1
COL1A1	Collagen, type 1, alpha 1
FBN	Fibrillin
COL15A1	Collagen, type 15, alpha 1
COL24A1	Collagen, type 24, alpha 1
GC	Guanine–cytosine
AT	Adenine–thymine
IL-6	Interleukin-6
PDPN	Podoalanin
CTSS	Cathepsin S
